# Are the calf and the thigh reliable alternatives to the arm for cuff non-invasive measurements of blood pressure?

**DOI:** 10.1186/cc9494

**Published:** 2011-03-11

**Authors:** K Lakhal, C Macq, S Ehrmann, T Boulain, X Capdevila

**Affiliations:** 1 CHU, Montpellier, France; 2 CHRU, Tours, France; 3 CHR, Orléans, France

## Introduction

Non-invasive measurement of blood pressure (NIBP) is widely used in the critically ill, the cuff being often placed on the calf or the thigh in case of contraindication for placing it on the arm (wounds, fracture, vascular access, and so forth) [1]. However, this common practice has never been validated. We assessed the reliability of NIBP at these different anatomic sites.

## Methods

Included: adult ICU patients carrying an arterial catheter. Excluded: mean arterial pressure (MAP) increase >5 mmHg during cuff inflation (inflation-induced pain); nonperception of the distal pulse despite the resolution of an eventual circulatory failure. For each site (arm, calf, thigh (if Ramsay score >4)), three pairs of NIBP and invasive measurements were respectively averaged. Patients in circulatory failure (MAP <65 mmHg and/or skin mottling and/or cathecholamine infusion) underwent a second set of measurements, after hemodynamic intervention (volume expansion and/or initiation and/or increase in catecholamine dosage). The agreement was assessed via a Bland-Altman analysis.

## Results

Ten patients were excluded and 11 NIBP measurements failed to display any figure: one patient for each site, eight others for the thigh only. Thus, 150 patients were analyzed (41 ± 26 years, BMI 26 ± 6, SAPS II 46 ± 18, Ramsay score = 5 or 6: 83%, mechanical ventilation 99%), comprising 79 patients with circulatory failure (MAP 70 ± 12 mmHg, norepinephrine (*n *= 62) 0.3 ± 0.3 μg/kg/minute, epinephrine (*n *= 2) 0.15 ± 0.14 μg/kg/minute). Absolute value of BP - for MAP measurement, NIBP performed better if the cuff was placed on the arm: bias/upper and lower limits of agreement (mmHg) of 3 ± 5/13/-6, 3 ± 8/18/-12 and 6 ± 7/20/-8 on the arm, the calf and the thigh, respectively. NIBP accuracy was similar in case of (mild) circulatory failure. Whatever the anatomic site, NIBP accuracy was better for MAP than for SAP or DAP. MAP changes - among the 57 patients with circulatory failure who underwent a second set of measurements after hemodynamic intervention, MAP changes (%) were better reflected when the cuff was placed on the arm, rather than on the calf or the thigh: 3 ± 5/12/-7, 3 ± 9/20/-14 and 3 ± 7/17/-10, respectively.

## Conclusions

For better reliability of MAP (and its changes) measurements, the cuff should be placed on the arm (if possible) rather than the thigh or the calf.
